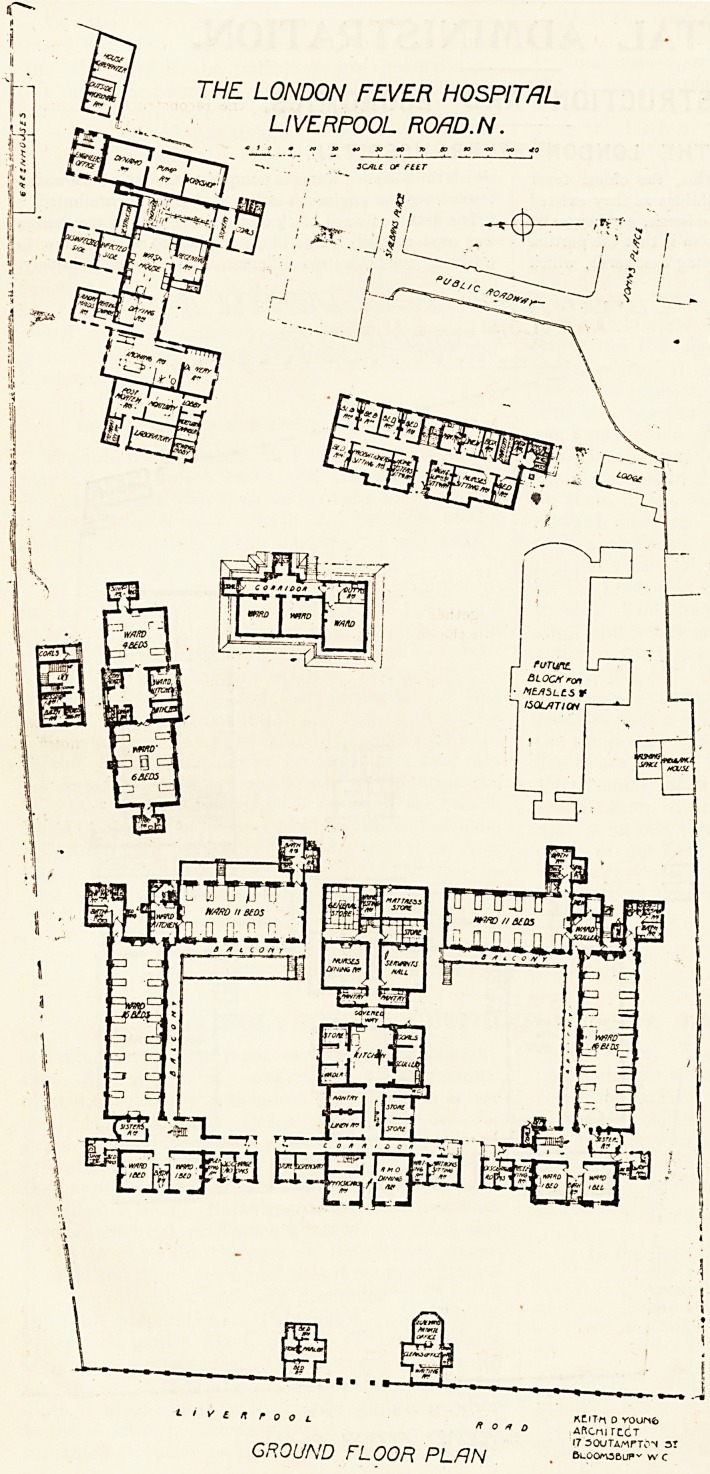# The London Fever Hospital

**Published:** 1908-08-29

**Authors:** 


### HOSPITAL ADMINISTRATION.

### CONSTRUCTION AND ECONOMICS.

### THE LONDON FEVER HOSPITAL.

The plans which we publish of this, the oldest fever hospital in the country, show the buildings as they existed up to the year 1880 and, with one omission, as they exist to-day. The omission on the latter plan is that the portion of the wing on the south side extending eastwards, which still remains, has been left out, and the outline of a projected block for measles and isolation shown in its place.

The original hospital consisted of all that is shown on the 1880 plan except the typhus wing. The rooms in the front building marked "private wards" were thrown together, and these were the scarlatina wards. The large wards marked "scarlet wards" were the typhus wards, as also were the wards marked "enteric."

The first step towards a general scheme for reconstruction was the erection in 1880 of the small isolation block. Up to that time there had been no provision for isolating doubtful cases or cases with complications or of such a nature as to render it inexpedient or dangerous to admit them to the general wards. This small block provided the needful means of isolation, and was the parent of many such buildings all over the country. It formed also the basis on which the Local Government Board model isolation block was designed.

The next step was the erection of the nursing home. Following this the Committee acquired additional land at the north-east angle of the site and erected thereon a boiler house, engine room, workshops, laundry, mortuary, and pathological laboratories. The whole of the hot water for heating and for draw-offs, the steam for cooking, sterilising, washing, and driving the electric light plant is generated in two boilers, one of which is equal to the whole work of the hospital. The electric light is produced at a cost very considerably less than that charged by the borough council. It has been possible to effect this by using up every pound of steam, allowing nothing to go to waste. In this block of buildings is a destructor for refuse of all kinds, an important feature in a fever hospital where no refuse of any sort can be sent outside the walls, and the disinfecting plant. There is also a water-softening plant just outside the boiler-house, from which the softened water is pumped to the top of the water-tower over the engineer's office, and thence distributed.

The detached ward block on the north side of the garden was next erected. This block is planned with a view to its being used for two different diseases simultaneously.

THE LONDON FEVER HOSPITAL LIVERPOOL Rd N.

AS IT EXISTED UP TO 1880

LIVERPOOL ROAD

GROUND FLOOR PLAN

GROUND FLOOR PLAN

The staircase, therefore, which connects the two floors is placed in a separate building, and communication between it and the upper floor is by way of an open bridge with a glass roof, which crosses the road between the two blocks. The ground floor of the staircase block contains also a set of discharging rooms.

The remodelling of the old hospital is the last work accomplished. The old main wards were double wards, with four rows of beds between the outside walls. This mode of planning is defective, inasmuch as efficient ventilation is impossible, and complaints of ill-effects from the want of proper ventilation were made from time to time by the medical staff. The sanitary offices also were most defective. There were no proper bathrooms, and there was no attempt at disconnecting these offices from the wards. These defects existed in an almost worse degree in connection with the private wards.

Plans had been prepared and had been considered by the committee for the complete rebuilding of the scarlatina wards and of the main administration building, but it was found impossible to embark on the large expenditure which these would have involved owing to the lack of funds and the difficulty in raising money for such a purpose. It became necessary, therefore, to devise a less ambitious scheme, which, while effecting the desired improvements, would not diminish to any serious extent the total number of beds.

The plan adopted provided for the demolition of one-half of each of the large double scarlatina wards, together with the sanitary offices and the one-story cross wards (marked on plan "enteric" wards). The remaining halves of the large wards were refloored with teak, the ceilings lowered, and the windows heightened, and new ward kitchens and sanitary offices added. A two-story block was erected on each side to take the place of the one-story cross ward. Wide balconies, on to which beds can be wheeled, are provided to all the wards, with an escape staircase to each block. The private wards, of which there are eight, four on each side, were altered, and provided with bathrooms and sanitary offices, and the floors relaid with teak. The inordinate height of the ceilings of these rooms (17 feet) was reduced, and the windows taken up to ceiling level.

The alterations to the female scarlatina wards involved the removal of part of the old wing, but though in the plan this block is shown to be entirely removed, a portion of it still remains, affording accommodation to measles cases. In the future, when funds admit, it is intended to remove this block and erect a new two-story block for measles and for isolation. The old boiler-house and laundry block has been swept away, and a new partly two-story block, containing dining-room and pantry for nurses, servants' hall and pantry, stores and testing laboratory, with bedrooms for servants on the upper floor, erected in its place. In the plan of the Nurses' Home the following corrections require to be noted. The front south bedroom is a pantry and the corresponding one at the north end is a sitting room for the matron's assistant. The room marked pantry on the right of the stairs is a bedroom and the room marked lavatory is a library.

For the present, therefore, the reconstruction scheme is at a standstill for want of the needful funds to carry it on. There remains to be done the new block for measles and isolation, the necessary extension of the nurses' home, and some much-needed repairs to the stonework of the main front.

The whole of the works have been planned and carried out under the hospital architect, Keith D. Young, F.R.I.B.A.

## Figures and Tables

**Figure f1:**
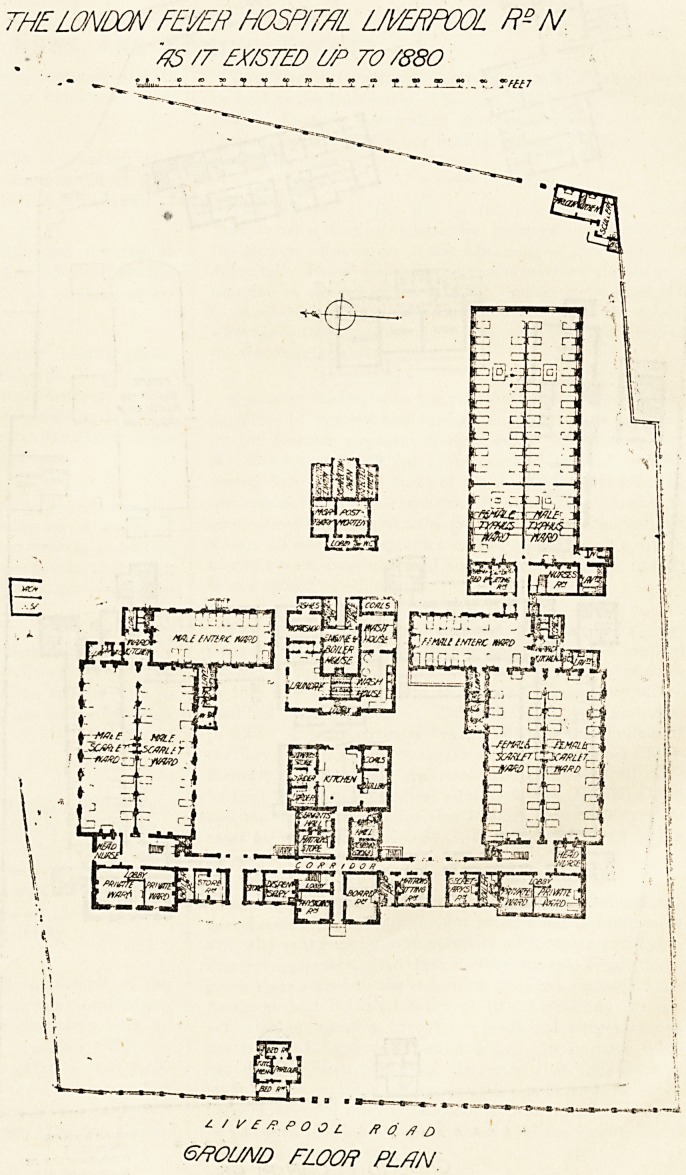


**Figure f2:**